# Magnetomitotransfer: An efficient way for direct mitochondria transfer into cultured human cells

**DOI:** 10.1038/srep35571

**Published:** 2016-10-21

**Authors:** Tanja Macheiner, Vera Heike Ingeborg Fengler, Marlene Agreiter, Tobias Eisenberg, Frank Madeo, Dagmar Kolb, Berthold Huppertz, Richard Ackbar, Karine Sargsyan

**Affiliations:** 1 Biobank Graz, Medical University of Graz, Austria; 2 Institute of Molecular Biosciences, NAWI Graz, University of Graz, Austria; 3 BioTechMed Graz, Graz, Austria; 4 Institute of Cell Biology, Histology and Embryology, Medical University of Graz, Austria; 5 Core Facility Ultrastructure Analysis/Center for Medical Research (ZMF), Medical University of Graz, Austria; 6 CBmed GmbH – Biomarker Research in Medicine, Graz, Austria

## Abstract

In the course of mitochondrial diseases standard care mostly focuses on treatment of symptoms, while therapeutic approaches aimed at restoring mitochondrial function are currently still in development. The transfer of healthy or modified mitochondria into host cells would open up the possibilities of new cell therapies. Therefore, in this study, a novel method of mitochondrial transfer is proposed by anti-TOM22 magnetic bead-labeled mitochondria with the assistance of a magnetic plate. In comparison to the passive transfer method, the magnetomitotransfer method was more efficient at transferring mitochondria into cells (78–92% vs 0–17% over 3 days). This transfer was also more rapid, with a high ratio of magnetomitotransferred cells and high density of transferred mitochondria within the first day of culture. Importantly, transferred mitochondria appeared to be functional as they strongly enhanced respiration in magnetomitotransferred cells. The novel method of magnetomitotransfer may offer potential for therapeutic approaches for treatment of a variety of mitochondria-associated pathologies, e.g. various neurodegenerative diseases.

Mitochondrial dysfunction arises through acquired or inherited mutations in mitochondrial DNA (mtDNA) or nuclear DNA as well as environmental causes such as infection or drug reaction^1^,^2^,^3^. Mitochondrial dysfunction plays also a key role in tumor progression, including metastasis and resistance to therapy^4^,^5^. Mitochondrial disease can develop from the early embryo period to adulthood and may manifest a wide variety of clinical symptoms from multiple organs. Typically, organs with high oxygen and energy requirements, such as heart, brain, and liver, are affected first^6^. Standard care focuses on treatment of the symptoms using e.g. antioxidants; however, such treatments often suffer from a lack of sufficient clinical data to support their general use^7^.

Potential therapies in development include gene therapy and cell-based therapies. In gene therapy, a gene is inserted into a cell for treatment of a disease. As an example, a clinical trial is currently in recruitment for gene therapy in Leber hereditary optic neuropathy, a disease caused by mutated mtDNA for which there is currently no effective therapy. The clinical trial in question uses “nuclear versions” of the mutated mitochondrial gene, with the cytoplasmically synthesized protein being directed to the mitochondria using a targeting sequence^8^.

Cell-based therapies are based on delivery of whole healthy mitochondria into diseased tissue. One approach is the correction of biochemical abnormalities in the blood after allogenic stem cell transplantation, which has been demonstrated in the clinic for mitochondrial neurogastrointestinal encephalomyopathy^9^. Alternatively, the healthy mitochondria can be transferred into diseased cells. This can be achieved through direct injection of isolated mitochondria into the diseased area. For example, *in-vivo* animal models demonstrate that isolated mitochondria, injected into ischemic heart tissue, are taken up by the cardiomyocytes and can lead to enhanced cardiac function^10^. Transfer can also be achieved through intercellular mitochondrial transfer. It has been observed that cells with defective or deleted mtDNA can be rescued through intercellular transfer of mitochondria from human stem cells^11^,^12^.

In order to avoid disadvantages of allogenic transplantation, healthy allogenic mitochondrial cells could be transferred into a patient’s own stem cells prior to autologous stem cell transplantation. For this, methodologies for the efficient and non-destructive isolation as well as subsequent transfer of mitochondria are required.

The standard procedure for isolation of mitochondria involves the rupturing of cells followed by centrifugation steps. Mitochondria, despite their large size and negative surface charge, can then be taken up by cells, through simple co-culturing^13^,^14^. Internalised mitochondria can enhance cell viability, although it is reported that the mitochondria disappear after approximately a week, with some mitochondria observed in the autophagosome. Evidence suggests that the mechanism for uptake into the cells could be through macropinocytosis, although this is debatable as it would imply that the mitochondria would have to escape the endosome to function^13^.

One of the main drawbacks of the standard approach is that it results in a crude cell extract containing both intact and damaged mitochondria, plus other organelle debris, which can be potentially harmful to the host cell^13^. Methodologies using anti-TOM22 magnetic beads have been shown to result in mitochondria isolates with increased purity and reproducibility from cells derived from mouse tissues^15^. It is therefore the aim of this study to investigate the use of magnetic beads for the improved isolation of mitochondria from human cells and furthermore, to investigate the potential benefits of a magnetic assisted transfer of mitochondria into host cells (referred to here as magnetomitotransfer) in comparison to standard passive co-culturing^11^,^16^.

## Results

### Quality of mitochondria isolated with magnetic beads

**Figure 1:**
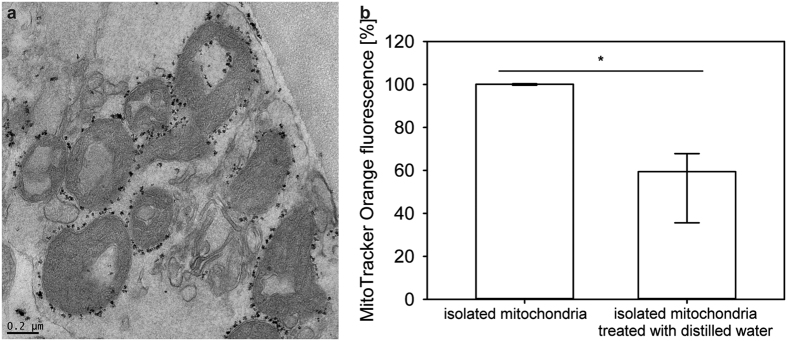
Quality of isolated mitochondria: (**a**) Electron microscopic evaluation of mitochondria after isolation procedure by magnetic labeling, with intact tubular shape and no evidence of significant swelling. The black dots on the outer mitochondrial membrane are the magnetic beads attached to the anti-TOM22 antibodies. (**b**) Statistically significant differences of mitochondrial activity measured by MitoTracker Orange in comparison between isolated mitochondria in storage buffer and in a solution in distilled water (given are medians with interquartile ranges and relevant significant difference is marked by an asterisk, n = 4, *P* values of <0.05 by Mann-Whitney U test).

In order to obtain mitochondria for transfer, we first isolated mitochondria from human fibroblasts using anti-TOM22 magnetic beads and subjected them to electron microscopy for quality-evaluation. The isolation procedure was performed ten times and resulted in morphologically intact mitochondria with tubular shape and no evidence of swelling. The magnetic bead-labeled anti-TOM22 antibodies remained attached to the mitochondrial membrane. Electron-microscopically investigated mitochondria yielded an average diameter of 0.4 μm (n = 100) after isolation (Fig. 1a).

Isolated mitochondria were stained with MitoTracker Orange, a dye that is accumulating in mitochondria dependent upon their membrane potential. Intact isolated mitochondria showed a statistically significant higher intensity of fluorescence in fluorescence reader analysis than mitochondria identically isolated but degraded with distilled water after isolation (Fig. 1b).

### Magnetomitotransfer efficiency outranges the efficiency of passive mitochondrial transfer

FITC labeled transferred mitochondria were detected within cells from both passively transferred and magnetomitotransferred cells. The magnetomitotransfer method was performed five times and showed a greater ratio of transferred to non-transferred MRC-5 fibroblasts compared to the passive transfer, and also yielded high transfer efficiencies on day 0 (83%), day 1 (92%), and day 2 (78%). Intracellular localization of magnetomitotransferred mitochondria could be verified by 3D analyses of the confocal microscopic scans (see Supplementary Fig. S1). In comparison, passively transferred mitochondria were only observed in the cells on day 1 and 2 with a transfer-ratio of 17% in each sample.

**Figure 2:**
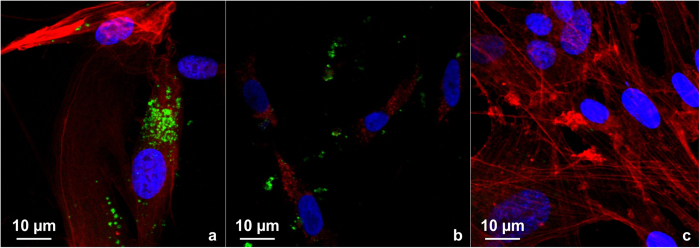
Laser scanning confocal (LSC) microscopically investigated mitochondrial transfer into MRC-5 fibroblasts: (**a**) LSC microscopic analysis of FITC-labeled mitochondria within magnetomitotransferred MRC-5 fibroblasts after one day in culture. (**b**) After passive transfer, only a few FITC labeled transferred mitochondria can be detected intracellular after one day of co-culture. (**c**) MRC-5 fibroblasts without magnetomitotransfer for negative control. Red: actin staining by Alexa Fluor 633 Phalloidin. Blue: DAPI staining of nuclei. Green: FITC-labeled mitochondria. Original magnification: ×650.

A trend for higher density of mitochondria in magnetomitotransferred cells was also observed (Fig. 2a,b), although this could not be quantified due to clumping of the signal; however, the signals were observed to become more scattered with time.

**Figure 3:**
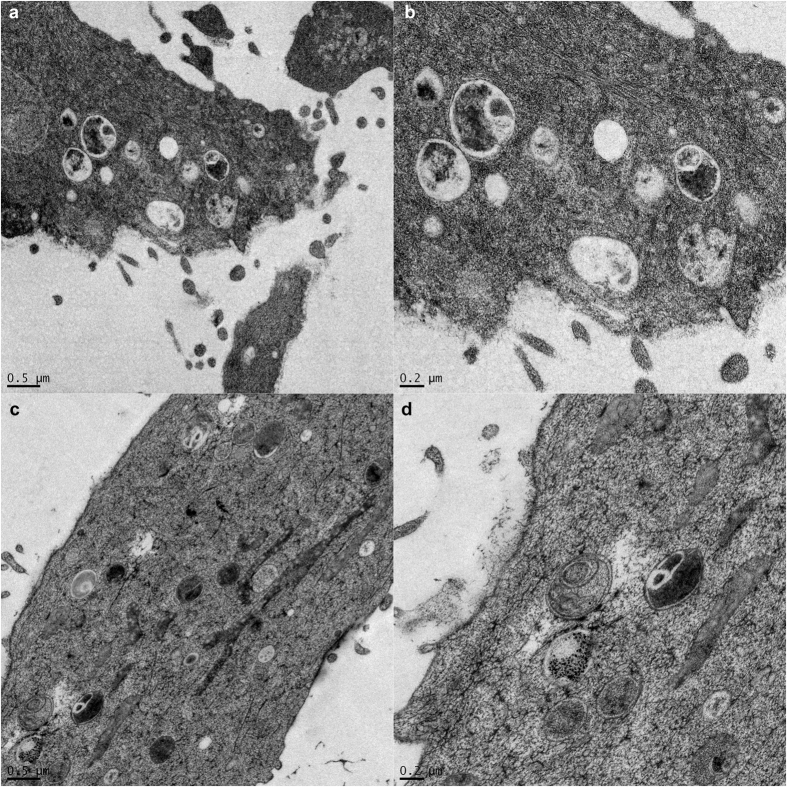
Electron microscopic analysis of MRC-5 fibroblasts after magnetomitotransfer revealed that intracellular membrane vesicles contain membranous structured contents and microbeads. Such structures are visible after two (**a,b**) and four days (**c,d**) of culture.

After magnetomitotransfer, transferred microbeads labeled mitochondria were found within intracellular membrane vesicles between day 2 and 4 (Fig. 3).

### Preservation of mitochondrial functions after magnetomitotransfer

**Figure 4:**
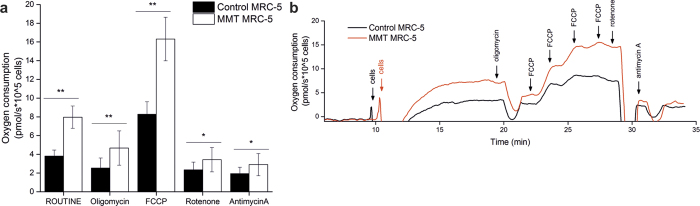
Magnetomitotransfer efficiently increases oxygen consumption of cultured MRC-5 fibroblasts: (**a**) Oxygen consumption assessed by high resolution respirometry of MRC-5 fibroblasts treated with microbeads only (control MRC-5) and magnetomitotransferred (MMT MRC-5) show a statistical significant higher oxygen consumption due to magnetomitotransfer. ROUTINE: routine respiration, Oligomycin: inhibition of ATP synthase, FCCP: maximal uncoupled respiration after stepwise titration of carbonyl cyanide p-trifluoromethoxyphenyl hydrazine (FCCP), Rotenone: inhibition of complex I, Antimycin A: inhibition of complex III. Data show means and standard deviations (n = 6, **P < 0.01, *P < 0.05; paired Student’s *t*-test, two-tailed). (**b**) Representative oxygraph recordings of MRC-5 fibroblasts treated only with microbeads and treated by magnetomitotransfer.

To address the functional consequence of successfully transferred intact mitochondria, we next subjected magnetomitotransferred MRC-5 fibroblasts to high-resolution respirometry. MRC-5 fibroblasts treated with microbead-labeled mitochondria exhibited at least two times increased oxygen consumption compared to MRC-5 fibroblasts treated only with microbeads in the magnetic field (Fig. 4). This elevated oxygen consumption was observed in routine respiration (i.e. respiration in culture media) as well as after addition of the mitochondrial uncoupler FCCP (carbonyl cyanide p-trifluoromethoxyphenylhydrazone) to assess the maximal respiratory capacity, suggesting the presence of an increased amount of functional mitochondria per cell after magnetomitotransfer. Inhibition of complex I by Rotenone and complex III by Antimycin A caused a robust reduction in oxygen consumption in both groups, confirming that mitochondrial derived respiration was detected (Fig. 4a,b).

### Cell growth after magnetomitotransfer

The real time x-CELLingence system was used for detection of cell growth for 70 h for magnetomitotransferred MRC-5 (n = 2) and MRC-5 treated with microbeads only as control (n = 1). The doubling time of magnetomitotransferred MRC-5 sample 1 was in average 71.6 h+/− 1.4 and of sample 2 79.2 h+/− 1.0. The doubling time of the control sample was 70.7 h+/− 0.7. Analyzing the growth rate of magnetomitotransferred MRC-5 fibroblasts and MRC-5 fibroblasts treated only with microbeads by x-CELLigence did not show any statistically significant differences (see Supplementary Fig. S2).

## Discussion

To the best of our knowledge, this study provides the first attempt for a magnetic mitochondria transfer in human cell culture. A method for transferring isolated mitochondria back into human cells facilitates autologous stem cell therapy approaches to mitochondrial diseases and hence, a reliable and efficient methodology is required. Therefore, this study investigated whether transfer of isolated mitochondria, back into human MRC-5 fibroblasts, with the assistance of a magnetic plate could be applied to increase transfer efficiency.

For this purpose, mitochondria exhibiting intact morphology and activity were successfully isolated from human MRC-5 fibroblasts, by labeling with anti-TOM22-bound magnetic beads^15^. These mitochondria we further subjected to the method of magnetomitotransfer. In comparison to the passive transfer method by simple co-culturing^13^, the magnetomitotransfer method was more efficient at transferring mitochondria into cells. This transfer was also more rapid, with a high ratio of magnetomitotransferred cells within the first day of culture. Furthermore, cells exposed to the magnetomitotransfer method also tended to show a high density of mitochondria per cell in comparison to the passive transfer method. Additionally, magnetomitotransferred mitochondria were detected intracellular by LSC as well as electron microscopy. Moreover, a higher mitochondrial activity of magnetomitotransferred MRC-5 fibroblasts after one day in culture was detected by oxygen consumption of the cells. The results confirmed that magnetomitotransfer significantly elevated the oxygen consumption in routine and maximal (after mitochondrial uncoupling) in MRC-5 fibroblasts.

However, magnetomitotransferred mitochondria were detected within intracellular membrane vesicles likewise with the passive transfer method, where internalized mitochondria were also identified in autophagosomes^13^. Therefore, the observed vesicle formation seems to be independent of the transfer method. Furthermore, the increased oxygen consumption of the magnetomitotransferred cells suggests that the transferred mitochondria within observed vesicles are intact and involved in ATP synthesis, at least at the period observed in here.

Nevertheless, further research is necessary for investigation of long-term effects as well as for the need of approaches to avoid intracellular vesicle formation or breaking up of these intracellular vesicles. A further consideration would be the assessment of the dose-dependent potential toxicity of magnetic beads to the recipient cells^17^, even if such microbeads are also used for magnetic stem cell selection and therapy without any apparent side effects for patients^18^.

Nevertheless, further experiments with lower microbead concentrations are required to test possible toxic effects of microbeads.

Biologically functional and stable transferred mitochondria, which contribute to the function of the recipient cells would e.g. enable internalization of healthy mitochondria into ovules carrying mutated mtDNA resulting human hereditary mitochondrial disorders^13^ or can be used for treatment of other diseases associated with mitochondrial dysfunction, such as myocardial infarction, diabetes mellitus or various liver diseases. Magnetomitotransfer is demonstrated to be a more rapid and efficient method, in terms of ratio of transferred to non-transferred cells as well as density of mitochondria transferred, in comparison to the traditional passive method. Furthermore, mitochondrial activity and intact morphology of isolated and magnetomitotransferred mitochondria could be maintained during magnetomitotransfer. In future, the magnetomitotransfer of mitochondria from other cells such as stem cells as well as the interaction of released mitochondria by tissue disruption on immune cells need to be further investigated^18^.

In conclusion, this study provides qualitative and quantitative evidence that the magnetomitotransfer of isolated mitochondria into MRC-5 fibroblasts paves the way for promising approaches and future novel treatment of diseases associated with mitochondrial dysfunction.

## Materials and Methods

For this study only commercially available MRC-5 cell line was used and methods were carried out in accordance with the approved guidelines by the local ethics committee of Medical University of Graz (26-312ex13/14).

### Isolation of mitochondria

Mitochondria were isolated from cells of the human fetal lung fibroblast cell line MRC-5 using a human Mitochondria Isolation Kit (Miltenyi Biotec). Cell lysates of 10^7^ cells were prepared by applying 1 ml lysis buffer and 18 strokes with a 26G needle. Cell lysates were incubated with anti-TOM22 microbeads for 1 h at 4 °C. Subsequently, magnetic separation of mitochondria from the cell lysate was carried out in a MACS Separator (Miltenyi Biotec) as for manufacturer’s procedures. Isolated mitochondria were centrifuged at 13,000 g for 2 min at 4 °C after which the supernatant was removed. Isolated mitochondria were further treated according to the requirements of the respective experiment.

### Assessment of isolated mitochondria

For the assessment of morphology, isolated mitochondria were analyzed by transmission electron microscopy. Isolated mitochondria were fixed in 2.5% (wt/vol) glutaraldehyde and 2% (wt/vol) paraformaldehyde in 0.1 M phosphate buffer, pH 7.4, for 2 h, postfixed in 2% (wt/vol) osmium tetroxide for 2 h at room temperature, dehydrated in graded series of ethanol and embedded in a TAAB epoxy resin (Gröpl).

Ultrathin sections (75 nm) were cut with a Leica UC 6 ultramicrotome and stained with lead citrate for 5 min and with uranyl acetate for 15 min. Images were taken using a FEI Tecnai G2 20 transmission electron microscope (FEI Eindhoven) with a Gatan ultrascan 1000 ccd camera. Acceleration voltage was 120 kV (FEI Tecnai 20 Transmission Electron Microscope).

For assessment of mitochondrial activity, fluorescence of MitoTracker^®^ Orange CM-H2TMRos (MitoTracker Orange, Life Technologies) was quantified. MitoTracker Orange is oxidized by oxygen in actively respiring cells^19^,^20^,^21^. Therefore, MitoTracker Orange solution (10 μl of a 100 μg/ml solution in dimethylsulfoxide per well in a 96-well plate) allows measuring the oxidative activity and was used to assess the viability of isolated mitochondria, prior to transfer. This was compared to control mitochondria disrupted by exposure to distilled water. The concentration of mitochondria was first standardized between samples by analysis of FITC labelling of anti-TOM22 microbeads. Fluorescence of MitoTracker Orange was quantified using a fluorescence reader (FLUOstar OPTIMA).

### Transfer of isolated mitochondria

Two methods for transfer of mitochondria were applied to MRC-5 fibroblast cultures.

#### Magnetomitotransfer

Isolated mitochondria from 10^7^ cells in 0.5 ml storage buffer were incubated with 2 μl Lipofectamine (Invitrogen LOT 1558640) for 10 min at 37 °C before adding to a confluent fibroblast monolayer in a single chamber slide (LAB-TEK Lot 1005222) chamber with 1.7cm^2^ for LSC microscopy or in cell culture flasks for growth experiments and oxygen consumption measurements. The chamber slides and the cell culture flasks were then placed on a magnetic plate (Conrad 503672 N35M, 1.21T, magnetic flux 79.5 × 10^−6^ Wb) for 20 min at room temperature that the applied mitochondria were pulled down into the cells by the magnetic field (observed cell mortality seems to be dependent on the used culture dishes and the used isolated mitochondria concentration since the cell mortality ratio was higher in chamber slides than in culture flasks (personal observation)). Lipofectamine was applied because of preliminary experiments, which showed cell damage as a result of magnetomitotransfer without Lipofectamine. For LSC microscopy, cells were subsequently incubated for up to five days at 37 °C in an incubator. As a negative control for growth experiments and oxygen consumption measurements fibroblasts were treated with anti-TOM22 microbeads only.

#### Transfer by co-culturing

In the second transfer methodology, the same protocol was applied as for magnetomitotransfer, with the exception that the cells were not applied to a magnetic plate. Isolated mitochondria from 10^7^ cells in 0.5 ml storage buffer were incubated with 2 μl Lipofectamine (Invitrogen LOT 1558640) for 10 min at 37 °C before adding to a confluent fibroblast monolayer in a single chamber slide (LAB-TEK Lot 1005222) chamber. The chamber slide was then left for 20 min at room temperature. Cells were subsequently incubated for up to two days at 37 °C.

### Assessment of mitochondria transferred cells

For analyses by laser scanning confocal (LSC) microscopy, 1.5 ml storage buffer (Miltenyi) and 15 μl ready-to-use FITC (Dako) were added to isolated mitochondria followed by vortexing and incubation for 10 min at room temperature. Finally, the mitochondria were centrifuged at 13,000 g for 2 min at 4 °C, the supernatant was removed and the mitochondria were resuspended in 0.5 ml storage buffer (Miltenyi Biotec). The isolated mitochondria were then used immediately for the transfer experiments. MRC-5 transferred fibroblasts on chamber slides were rinsed in PBS and fixed in 3.7% formaldehyde for 3 min and after rinsing in PBS again by −20 °C cooled acetone. After repeated washing in PBS 5 μl Phalloidin stock solution (Alexa Fluor 633) was diluted to 200 μl with PBS per slide for actin staining and together with 20 μl Hoechst solution (5 μl Hoechst 33342 stock solution in 10 ml PBS) for nuclear counterstain incubated for 30 min in a moisture chamber at room temperature. For analyses by LSC microscopy a Zeiss LSM 510 META microscope and Zeiss Zen software were used.

For determining the intracellular location and fate of the transferred mitochondria, cells were analyzed by transmission electron microscopy using high pressure freezing. Therefore, cells were loaded and frozen using 2000 bar under liquid nitrogen conditions within milliseconds. Freezing was followed by freeze substitution in acetone by adding 2% osmium tetroxide (OsO4) and 0.2% UAc were added at temperatures below −70 °C. The water in form of ice in cells was replaced by substitution media and afterwards embedded in Epoxy resin. Afterwards specimens were treated as described in Assessment of isolated mitochondria.

### Measurement of oxygen consumption after magnetomitotransfer

One day after magnetomitotransfer, MRC-5 cells were treated with Accutase (Sigma-Aldrich) and resuspended in 0.6 ml fresh DMEM (Sigma-Aldrich) yielding a cell density of ~2 × 10^5^–4 × 10^5^ cells/ml. Cell densities were determined by CASY Cell Counter technology (Roche Applied Sciences) and used to calculate relative oxygen consumption per cell. 2 ml of cell suspension were applied to high-resolution respirometry assayed at 37 °C with an Oxygraph-2k (Oroboros Instruments) respirometer according to manufactures recommendations and similar to published procedures^22^. Briefly, after initially recording routine respiration (ROUTINE) the ATP synthase inhibitor oligomycin (2.5 μM) was added, followed by titration with FCCP (0.25 μM steps) leading to an uncoupled state. Finally, complex I and complex III activities were inhibited by rotenone (2.5 μM) and antimycin A (2.5 μM). Magnetomitotransferred cells were always processed in parallel to control cells (i.e. cells treated with microbeads only). Therefore, a paired Student’s t-test (two-sided) was used to detect statistically significant differences between groups after confirming normality and homogeneity of variance.

### Determination of cell growth

The impedance-based x-CELLingence system (ACEA Bioscience Inc.) was placed at 37 °C in a humidified 5% CO_2_ incubator. MRC-5 fibroblasts, including two samples magnetomitotransferred samples and one control sample, (5 × 10^3^) were seeded in each well and placed in the x-CELLingence system and proliferation was measured for 70 h.

## Additional Information

**How to cite this article**: Macheiner, T. *et al*. Magnetomitotransfer: An efficient way for direct mitochondria transfer into cultured human cells. *Sci. Rep.* **6**, 35571; doi: 10.1038/srep35571 (2016).

## Supplementary Material

Supplementary Information
