# Supplementary material for: Magnetomitotransfer: An efficient way for direct mitochondria transfer into cultured human cells
Source: Sci Rep. 2016 Oct 21;6:35571. doi: 10.1038/srep35571 (PMC5073296; doi:10.1038/srep35571)
Supplement: Supplementary Information [file srep35571-s1.pdf]

Magnetomitotransfer: An efficient way for direct mitochondria transfer into cultured human cells

Tanja Macheiner, Vera Heike Ingeborg Fengler, Marlene Agreiter, Tobias Eisenberg, Frank Madeo, Dagmar Kolb, Berthold Huppertz, Richard Ackbar, Karine Sargsyan

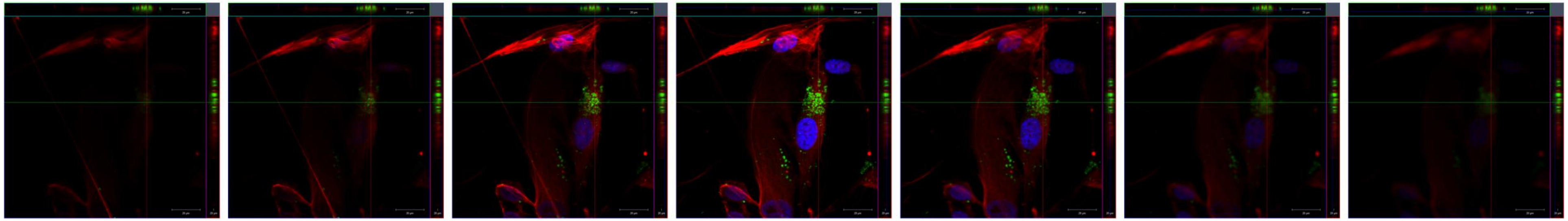

Fig S1. 3D analyses of LSC microscopic scans: The cell level series of a magnetomitotransferred MRC-5 fibroblast including the signal analyses on the edges shows the intracellular green signal of magnetomitotransferred mitochondria (FITC-labeled mitochondria).

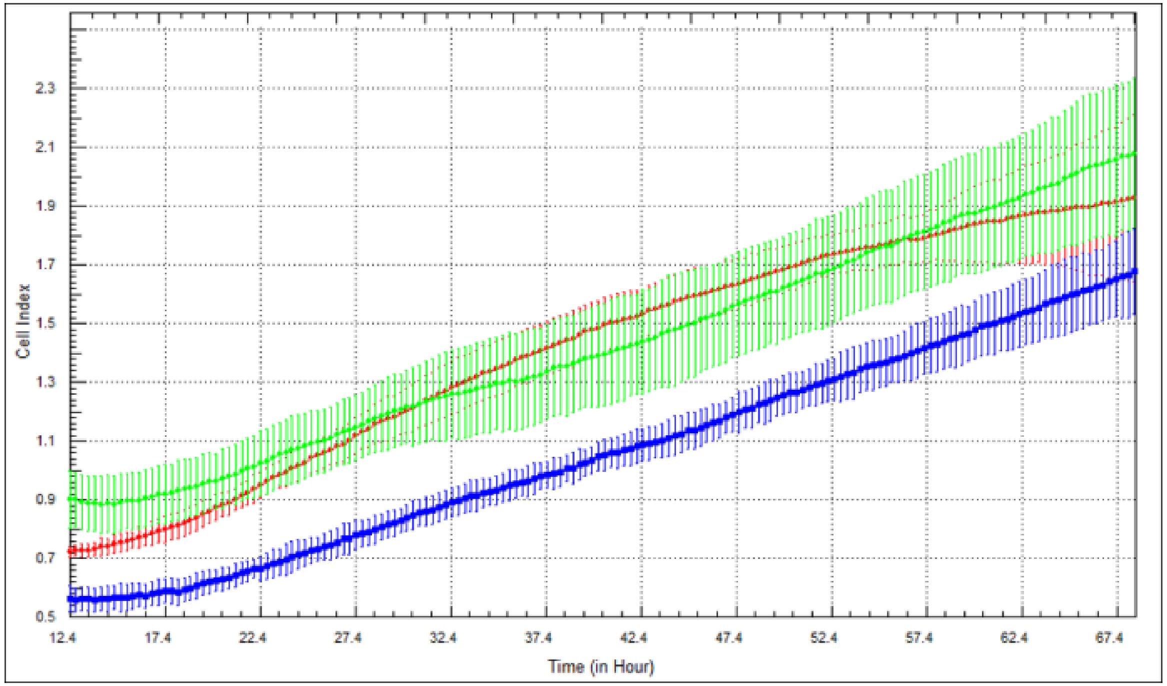

Fig S2. The growth and survival abilities of the MRC-5 fibroblasts after magnetomitotransfer were determined using an x-CELLingence biosensor system. The number of cells is represented as the cell index (y-axis). The experiment shows no statistical significant differences between magnetomitotransferred MRC-5 fibroblasts (red and green) and the control MRC-5 fibroblasts (blue), which were treated with microbeads only.
